# Establishment of *Amblyomma maculatum* Ticks and *Rickettsia parkeri* the Northeastern United States 

**DOI:** 10.3201/eid3010.240821

**Published:** 2024-10

**Authors:** Goudarz Molaei, Noelle Khalil, Carmen J. Ramos, Christopher D. Paddock

**Affiliations:** Yale School of Public Health, New Haven, Connecticut, USA (G. Molaei);; Connecticut Agricultural Experiment Station, New Haven (G. Molaei, N. Khalil);; Centers for Disease Control and Prevention, Atlanta, Georgia, USA (C.J. Ramos, C.D. Paddock)

**Keywords:** vector-borne infections, rickettsia, *Amblyomma maculatum*, *Rickettsia parkeri*, tickborne infections, bacteria, arboviruses, ticks, United States

## Abstract

We document a case of *Rickettsia parkeri* rickettsiosis in a patient in Connecticut, USA, who became ill after a bite from a Gulf Coast tick (*Amblyomma maculatum*). We used PCR to amplify *R. parkeri* DNA from the detached tick. The patient showed a 4-fold rise in IgG reactive with *R. parkeri* antigens.

Native and invasive tick species pose serious public health concerns in the United States, particularly in northeastern states. Recent and rapid expansion of the lone star tick (*Amblyomma americanum*) into ranges with pervasive blacklegged tick (*Ixodes scapularis*) populations has increased the number of recognized tickborne pathogens that circulate in that densely populated region. In addition to *Borrelia burgdorferi*, the causative agent of Lyme disease, >7 additional tickborne pathogens are now endemic to the northeastern United States: *Ehrlichia chaffeensis*, *Ehrlichia ewingii*, Heartland virus, *Anaplasma phagocytophilum*, *Borrelia miyamotoi*, *Babesia microti*, and Powassan virus (*1*). Multiple factors, including climate change and anthropogenic modifications to the environment, have affected rapid expansion of the ranges of medically relevant tick species and associated pathogens. That expansion has been reflected by dramatic increases in the numbers of reported cases of tickborne diseases in the northeastern United States since the beginning of the 21st Century (*1*). 

The Gulf Coast tick (*Amblyomma maculatum*) was first identified in the United States in 1844. As recently as the middle of the 20th Century, the tick’s range was restricted predominantly to coastal regions of states bordering the Gulf of Mexico as far west as Texas and the southern Atlantic coast only as far north as southern North Carolina (Figure 1) (*2**,**3*). Established *A. maculatum* tick populations now exist in states hundreds of miles inland (Arkansas, Missouri, Kentucky, Illinois, Indiana) and along the Atlantic coast as far north as Connecticut (*4**–**9*). Migratory grassland birds serve a crucial role in the spread of Gulf Coast ticks to locations in central and northern states that possess favorable environmental conditions for the tick’s survival (*2*,*8*). 

**Figure 1:**
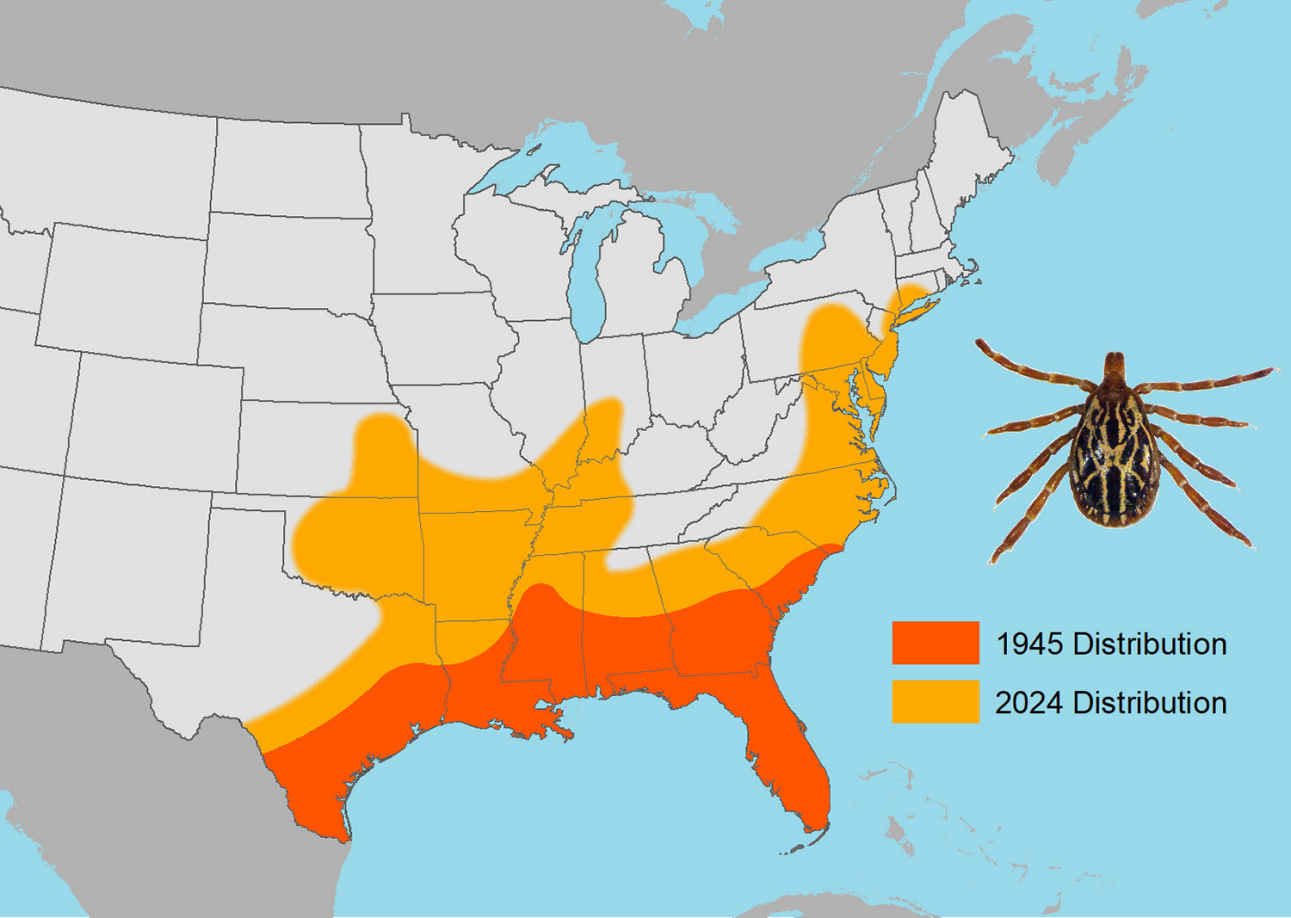
Generalized distributions of the Gulf Coast tick, *Amblyomma maculatum* (pictured), a human-biting tick species newly established in the northeastern United States, in 1945 compared with 2024. Establishment was defined as >6 ticks of the same life stage identified within a 12-month period or ticks of >1 life stage identified within a 12-month period. Data from references *2*–*9*, https://www.dep.pa.gov/Business/ProgramIntegration/Vector-Management/Ticks/Pages/default.aspx, and https://www.in.gov/health/idepd/zoonotic-and-vectorborne-epidemiology-entomology/vector-borne-diseases/tick-borne-diseases/amblyomma-maculatum-gulf-coast-tick/#Geographic_Distribution.

The Gulf Coast tick was relatively unknown and infrequently studied until recognition of *Rickettsia parkeri* spotted fever rickettsiosis in 2004 (*2*). In contrast to its more widely recognized cousins, blacklegged and lone star ticks, which prefer predominantly woodland habitats, Gulf Coast ticks favor grassland habitats. During the past 250 years, huge swathes of native grasslands and savannahs in the eastern United States have been transformed into agricultural areas and rangeland, creating habitats no longer favorable for Gulf Coast ticks. Paradoxically, contemporary reclamation of native grasslands through conservation efforts in the northeastern United States might have inadvertently led to establishment of Gulf Coast ticks in that region. The recent discovery of established populations of Gulf Coast ticks in reclaimed grasslands at the former Freshkills landfill on Staten Island, New York, is a salient example of this phenomenon (*7*,*8*). Of note, restored grassland habitats often occur near or within shorelines, parks, and wildlife areas proximate to and frequented by persons residing in densely populated metropolitan areas. 

The Gulf Coast tick is the principal vector of *R. parkeri*, a pathogen that causes a rickettsiosis similar to but milder than Rocky Mountain spotted fever (*2*). Rates of *R. parkeri* infection are as high as 56% among questing adult Gulf Coast ticks in some regions, and 23%–53% of adult specimens obtained in Connecticut, New York, and New Jersey are infected (*6**–**9*). Gulf Coast ticks readily bite humans, posing another risk for tickborne disease in northeastern United States, particularly among persons who reside and recreate near or within New York, New York; New Haven, Connecticut; Newark, New Jersey; and Philadelphia, Pennsylvania, where recently established tick populations have been identified in areas where human infections with this pathogen had not been previously documented (Figure 1). 

In August 2023, a 29-year-old woman discovered a tick attached to the nape of her neck after visiting a beach in Fairfield County, Connecticut. The tick (Figure 2, panels A, B) was attached for ≤4 hours before it was removed. Within 3 days, a small, erythematous, crusted lesion with a smaller satellite papule developed at the bite site (Figure 2, panel C), after which the patient experienced chills, fatigue, cervical lymphadenopathy, myalgia, severe headache, and mild confusion. Approximately 10 days later, several small erythematous macules developed on her arm and legs (Figure 2, panel D). The patient recovered rapidly after treatment with doxycycline. We performed PCR to amplify *R. parkeri* DNA from the detached tick; indirect immunofluorescence antibody assay results of patient serum samples revealed IgG reactive with antigens of *R. parkeri* at titers of <32 at 15 days and 256 at 24 days after illness onset (Appendix). 

**Figure 2:**
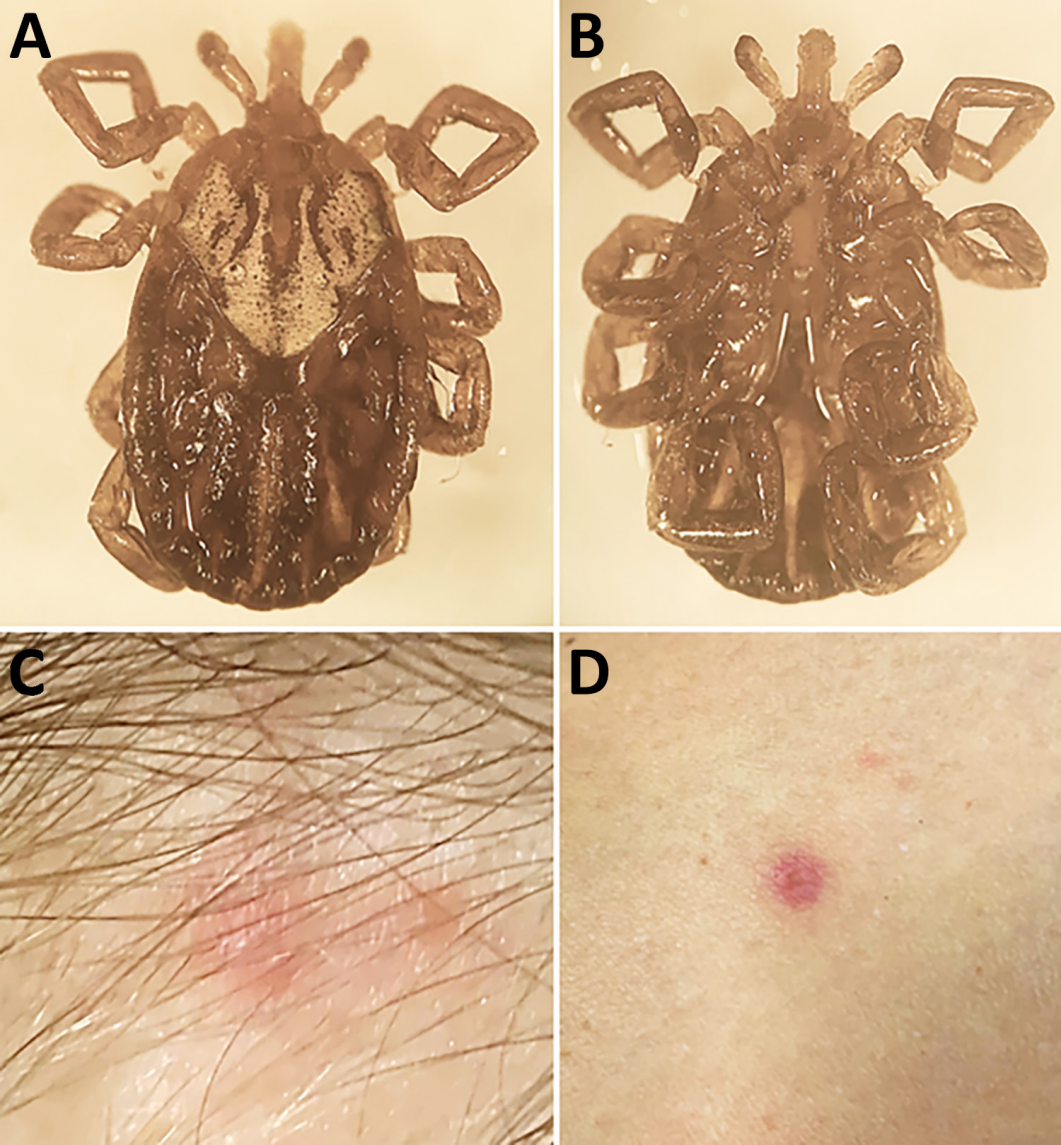
Biting *Amblyomma maculatum* tick removed from a woman in Connecticut, USA, and signs of *Rickettsia parkeri* rickettsiosis. A, B) Dorsal (A) and ventral (B) images of the tick. C, D) A small, erythematous, crusted lesion with a smaller satellite papule that developed at the bite site (C) and 1 of several small erythematous macules that developed on her arm and legs (D).

Because of morphologic similarities between Gulf Coast ticks and American dog ticks (*Dermacentor variabilis*, the principal vector of Rocky Mountain spotted fever in the northeastern United States), the 2 species can be misidentified. Because most tick species are associated with a unique suite of pathogens, it is critical to improve regional capacity for accurately detecting and identifying specific ticks and the pathogens they transmit in the northeastern United States, an area already endemic for Lyme disease, hard tick relapsing fever, Rocky Mountain spotted fever, ehrlichiosis, anaplasmosis, and Powassan virus infections (*10*). The rapidly changing dynamics and evolving risks of tickborne diseases across this region reinforce the need for awareness of and education on tick bite prevention strategies, including using repellents registered with the Environmental Protection Agency and performing regular, thorough tick checks after exposure to tick-infested areas. 

Appendix Additional information about migration of Gulf Coast ticks and *Rickettsia parkeri* rickettsiosis into the northeastern United States.
