# Supplementary material for: Establishment of Amblyomma maculatum Ticks and Rickettsia parkeri the Northeastern United States
Source: Emerg Infect Dis. 2024 Oct;30(10):2208–11. doi: 10.3201/eid3010.240821 (PMC11431899; doi:10.3201/eid3010.240821)
Supplement: Appendix — Additional information about migration of Gulf Coast ticks and Rickettsia parkeri rickettsiosis into the northeastern United States. [file 24-0821-Techapp-s1.pdf]

Article DOI: https://doi.org/10.3201/eid3010.240821

EID cannot ensure accessibility for supplementary materials supplied by authors. Readers who have difficulty accessing supplementary content should contact the authors for assistance.

Migration of *Amblyomma maculatum* Ticks and *Rickettsia parkeri* into the Northeastern United States

Appendix

Additional Methods

Initial examination of the tick at the Connecticut Agricultural Experiment Station Tick Testing Laboratory using a dissecting microscope and a standard taxonomic key (*1*) revealed that the tick was a female *Amblyomma maculatum*. To corroborate morphological identification, DNA was extracted from the tick specimen using DNAzol BD (Molecular Research Center, https://www.mrcgene.com) as previously described (*2*). PCR assay was conducted to amplify a portion of the ribosomal internal transcribed spacer 2 (*ITS2*) region using the following primer pair: 5′–CGAGACTTGGTGTGAATTGCA–3′ (forward) and 5′–TCCCATACACCACATTTCCCG–3′ (reverse) (*3*). A Taq PCR Core Kit (QIAGEN, https://www.qiagen.com) and Applied Biosystems Veriti Thermal Cycler (ThermoFisher Scientific, https://www.thermofisher.com) were used to conduct PCR assays consistent with the manufacturer’s instructions. PCR amplicons were purified using the QIAquick PCR purification kit (QIAGEN), and double stranded sequencing was performed at the Keck DNA Sequencing Facility, Yale University (New Haven, Connecticut, USA). Sequences were subsequently annotated using ChromasPro version 2.1.8 (Technelysium, https://technelysium.com.au) and submitted to the NCBI GenBank to compare with available sequences in the database. The specimen was determined to be *A. maculatum* based on 100% pairwise identity with several sequences of that tick species in GenBank. The sequence was later submitted to GenBank (accession no. OR625121.1).

A PCR assay was performed to amplify a 645-basepair portion of a rickettsial outer membrane protein gene (*omp*A) using the following primer pair: 5′–

ATGGCGAATATTTCTCCAAAA–3′ (forward) and 5′–ATTACCTATTGTTCCGTTAATGGCA–3′ (reverse) (*4*). PCR and sequencing of the purified amplicons were performed as described above. The annotated sequence was identified as *Rickettsia parkeri* based on >99% percent identity with several sequences belonging to this species in the GenBank database. The sequence was subsequently submitted to GenBank (accession no. OR517310.1).

An indirect immunofluorescence antibody (IFA) assay to detect IgG antibodies reactive with *Rickettsia parkeri* antigens was performed at the Centers for Disease Control and Prevention, as described previously (*5*).

References

1. Keirans JE, Litwak TR. Pictorial key to the adults of hard ticks, family Ixodidae (Ixodida: Ixodoidea), east of the Mississippi River. J Med Entomol. 1989;26:435–48. PubMed https://doi.org/10.1093/jmedent/26.5.435

2. Khalil N, Dugas KD, Cantoni JL, Stafford KC, Molaei G. Anomalous morphologies in *Ixodes scapularis* feeding on human hosts. Ticks Tick Borne Dis. 2022;13:101993. PubMed https://doi.org/10.1016/j.ttbdis.2022.101993

3. Chitimia L, Lin RQ, Cosoroaba I, Braila P, Song HQ, Zhu XQ. Molecular characterization of hard and soft ticks from Romania by sequences of the internal transcribed spacers of ribosomal DNA. Parasitol Res. 2009;105:907–11. PubMed https://doi.org/10.1007/s00436-009-1474-1

4. Lee KM, Choi YJ, Shin SH, Choi MK, Song HJ, Kim HC, et al. Spotted fever group rickettsia closely related to *Rickettsia monacensis* isolated from ticks in South Jeolla province, Korea. Microbiol Immunol. 2013;57:487–95. PubMed https://doi.org/10.1111/1348-0421.12062

5. Yaglom HD, Casal M, Carson S, O’Grady CL, Dominguez V, Singleton J Jr, et al. Expanding recognition of *Rickettsia parkeri* rickettsiosis in southern Arizona, 2016–2017. Vector Borne Zoonotic Dis. 2020;20:82–7. PubMed https://doi.org/10.1089/vbz.2019.2491
